# Community-based type 2 diabetes care by lay village health workers in rural Lesotho: protocol for a cluster-randomized trial within the ComBaCaL cohort study (ComBaCaL T2D TwiC)

**DOI:** 10.1186/s13063-023-07729-8

**Published:** 2023-10-24

**Authors:** Felix Gerber, Ravi Gupta, Thabo Ishmael Lejone, Thesar Tahirsylaj, Tristan Lee, Maurus Kohler, Maria Ines Haldemann, Fabian Räber, Mamakhala Chitja, Molulela Manthabiseng, Makhebe Khomolishoele, Mota Mota, Matumaole Bane, Pauline Mamorontsane Sematle, Retselisitsoe Makabateng, Madavida Mphunyane, Sejojo Phaaroe, Dave Basler, Kevin Kindler, Eleonora Seelig, Matthias Briel, Frédérique Chammartin, Niklaus Daniel Labhardt, Alain Amstutz

**Affiliations:** 1 Department of Clinical Research, Division of Clinical Epidemiology, University Hospital Basel, Basel, Switzerland; 2 University of Basel, Basel, Switzerland; 3 Swiss Tropical and Public Health Institute, Allschwil, Switzerland; 4 SolidarMed, Maseru, Lesotho; 5 Ministry of Health Lesotho, Maseru, Lesotho; 6 Faculty of Business, Economics and Informatics, University of Zurich, Zürich, Switzerland; 7 Clinic of Endocrinology, Diabetology and Metabolism, University Hospital Basel, Basel, Switzerland; 8 Department of Health Research Methods, Evidence, and Impact, McMaster University, Hamilton, Canada

**Keywords:** Type 2 diabetes, Community-based care, Village health workers, Community health worker, Clinical decision support system, Non-communicable diseases, Sub-Saharan Africa, Lesotho

## Abstract

**Background:**

Type 2 diabetes (T2D) poses a growing public health burden, especially in low- and middle-income countries (LMICs). Task-shifting to lay village health workers (VHWs) and the use of digital clinical decision support systems (CDSS) are promising approaches to tackle the current T2D care gap in LMICs. However, evidence on the effectiveness of lay worker-led T2D care models, in which VHWs initiate and monitor drug treatment in addition to community-based screening and referral services, is lacking.

**Methods:**

We are conducting a cluster-randomized trial nested within the Community-Based Chronic Disease Care Lesotho (ComBaCaL) cohort study (NCT05596773) using the trial within cohort (TwiC) design to assess the effectiveness of a VHW-led, CDSS-assisted T2D care model in rural Lesotho. Participants are non-pregnant members of the ComBaCaL cohort study with T2D. The ComBaCaL cohort study is conducted in approximately 100 villages in two rural districts in Lesotho and is managed by trained and supervised VHWs. In intervention villages, VHWs offer a community-based T2D care package including lifestyle counselling, first-line oral antidiabetic, lipid-lowering, and antiplatelet treatment guided by a tablet-based CDSS to participants who are clinically eligible, as well as treatment support to participants who prefer or clinically require facility-based T2D care. In control clusters, all participants will be referred to a health facility for T2D management. The primary endpoint is the mean glycosylated haemoglobin (HbA1c) 12 months after enrolment. Secondary endpoints include the 10-year risk for cardiovascular events estimated using the World Health Organization risk prediction tool.

**Discussion:**

The trial was launched on May 13, 2023, and has enrolled 226 participants at the date of submission (October 6, 2023). To our knowledge, the trial is the first to assess task-shifting of T2D care to VHWs at the community level, including the prescription of first-line antidiabetic, lipid-lowering, and antiplatelet medication in sub-Saharan Africa, and will thus provide the missing evidence on the effectiveness of such a T2D care model in this setting. The study is operating within the established Lesotho VHW programme. Similar community health worker programmes which exist across sub-Saharan Africa may benefit from the findings.

**Trial registration:**

ClinicalTrials.gov NCT05743387. Registered on February 24 2023.

**Supplementary Information:**

The online version contains supplementary material available at 10.1186/s13063-023-07729-8.

## Introduction

Globally, 11% of the adult population or 536.6 million individuals were estimated to be living with diabetes in 2021. By 2045, this number is expected to increase to 783.2 million [1]. Four out of five people affected by diabetes are living in low- and middle-income countries (LMICs) [2]. Over 90% of all diabetes cases are due to type 2 diabetes (T2D), which is also the main driver of the projected increase in the overall diabetes cases [3]. The increase in T2D prevalence is caused by ageing populations and changing lifestyles with decreasing levels of physical activity, higher caloric diets, and associated obesity [4]. Currently, only half of the people living with diabetes are aware of their condition [3, 5]. The risk for diabetes to remain undetected and untreated is significantly higher in LMICs than in high-income countries, and so is the risk for early complications [2, 6, 7]. At the same time, the management of diabetes complications is costly, and access to quality services for complications is limited in most LMICs [8]. Therefore, prevention via risk factor control and adequate antidiabetic treatment before the onset of complications is essential for an effective burden reduction [9, 10]. Setting-specific, affordable, and scalable solutions are needed to tackle the growing diabetes burden in LMICs [10].

Capacitating lay village health workers (VHWs) to deliver essential services at the community level is a promising approach to improve access to and outcomes of diabetes care in LMICs [11–16], especially in sub-Saharan Africa, where most health systems face a substantial shortage of professional healthcare workforce [17]. Screening, education, and self-management support interventions by VHWs at the community level have been tested successfully, but it remains unclear whether such interventions are sufficiently effective and scalable to close the existing treatment gap [10, 14, 16, 18, 19].

We developed a VHW-led care model for people living with T2D in rural Lesotho, based on a local non-communicable disease (NCD) prevalence survey and burden assessment [20], a scoping literature review [18], and the Community-based chronic disease care Lesotho (ComBaCaL) pilot cohort study. In this care model, VHWs provide first-line management for T2D, including oral antidiabetic, lipid-lowering, and antiplatelet treatment as well as lifestyle counselling at the community level, assisted by the ComBaCaL app, a tailored, tablet-based, digital clinical decision support system (CDSS).

We aim to evaluate the effectiveness and safety of this care model in a cluster-randomized trial embedded in the ComBaCaL cohort study (NCT05596773; www.combacal.org).

## Methods

### Setting

The ComBaCaL cohort study is conducted in 103 randomly selected rural villages in the two districts Butha-Buthe and Mokhothlong in Lesotho, a small, landlocked, high-altitude country encircled by South Africa. In each ComBaCaL village, one lay VHW has been selected by the village population in a participatory process according to the Lesotho Ministry of Health (MoH) Village Health Program policy [21]. Lesotho is a typical example of an African LMIC where a developing health system is facing the double burden of the still highly prevalent infectious diseases HIV/AIDS and tuberculosis in combination with a rapidly spreading NCD epidemic [2, 20, 22, 23]. In the Lesotho health system, VHWs play an important role in linking the community to facility-based health services and have effectively contributed to the improved control of HIV/AIDS, especially in remote rural areas [21, 24, 25].

### Design and hypothesis

We are conducting a 1:1 cluster-randomized, open-label trial nested within the ComBaCaL cohort study following a trial within cohort (TwiC) design [26, 27]. Our trial estimand and hypothesis is that offering community-based, VHW-led, CDSS-assisted T2D care in rural Lesotho is superior regarding glycosylated haemoglobin (HbA1c) levels (mean difference) 12 months after enrollment compared to offering facility-based T2D care among non-pregnant adults with uncomplicated (taking no or only one oral antidiabetic drug), uncontrolled (fasting blood glucose (FBG) ≥ 7 mmol/l) T2D who were still alive and did not move out of their village irrespective of the uptake of the intervention, T2D treatment adherence, and adverse events. The SPIRIT reporting guidelines were used to develop and report this protocol [28].

### Eligibility and consent procedure

Participants for this trial are recruited among the ComBaCaL cohort population, which includes all inhabitants of the randomly selected ComBaCaL villages who gave informed consent to participate in the ComBaCaL cohort study and to be randomly selected for nested TwiCs [26, 27]. No written consent for the TwiC itself is asked. Participants in the control group are followed according to the standard of care in the ComBaCaL cohort and participants in the intervention group are offered the intervention which they can accept or refuse. The participant information materials and the consent forms of the ComBaCaL cohort study (covering the nested TwiCs) are available from the corresponding author upon request. As per cohort procedures (outlined in the cohort study protocol), all adult ComBaCaL cohort participants with a body mass index (BMI) of 25 kg/m^2^ or above or aged 40 years or older are screened for T2D by their VHW according to a standardized diagnostic algorithm incorporated in the tablet-based ComBaCaL app. All non-pregnant adult participants of the ComBaCaL cohort study with T2D, defined as reporting intake of antidiabetic medication or being newly diagnosed during screening, are eligible for this TwiC. Following the TwiC design, participants in the intervention group may accept or refuse the intervention services by the VHW (see below) or else be referred to the responsible health facility for further care.

### Randomization and blinding

Half of the ComBaCaL cohort villages are randomly allocated to the intervention group by a statistician not involved in the study. The random allocation is stratified by district (Butha-Buthe versus Mokhothlong) and access to health facilities (easy versus difficult access, defined as needing to cross a mountain or river or travel > 10 km to the nearest health facility). VHWs who are enrolling participants, providing the intervention, and collecting secondary endpoint data are not blinded to the intervention. The primary endpoint (HbA1c) is a blood test conducted by the study staff not directly involved in the delivery of the intervention. Due to the cluster-level randomization and TwiCs approach, participants are blinded to the allocation meaning that participants in the control villages are not aware of the intervention being implemented in other villages.

### Trial intervention

In intervention villages, VHWs offer a community-based T2D care package that includes lifestyle counselling, lipid-lowering (statin) and antiplatelet (aspirin) treatment for eligible participants, and first-line antidiabetic (metformin) treatment for participants with uncomplicated T2D and treatment support with regular check-ups for participants with complicated T2D, which is defined as not reaching sufficient blood sugar control with metformin alone, thus requiring insulin or the addition of another oral antidiabetic medication. Guidance for treatment initiation, drug prescription, counselling, and monitoring is provided via the ComBaCaL app according to algorithms based on international guidelines for primary healthcare-level management of T2D [29, 30] and the current Lesotho Standard Treatment Guidelines [31]. All activities conducted by VHWs in the communities, including counselling and drug prescription, are captured in the same application. Supervising study staff monitors all activities in a web version of the application, and VHWs may request support from supervising study staff or routine healthcare professionals at the responsible health facility, if needed. In case of complicated disease, for example, if treatment targets are not reached with metformin alone, unclear diagnosis, potential contraindications, side effects, or the presence of clinical alarm signs or symptoms, the ComBaCaL app automatically suggests referring participants to the closest health facility for further management. Participants are free to accept or refuse the services offered by the VHW at any time. Participants refusing VHW-led services are referred to the responsible health facility for further management with two-monthly checks by the VHW at the community level.

In control villages, VHWs refer all participants found eligible during the screening to the responsible health facility for T2D care. VHWs will conduct a check-up with repeated referral (if required) 6 months after enrolment with no further services provided at the community level.

### Endpoints

The selection of endpoints is based on the International Consortium for Health Outcomes Measurements’ data collection reference guide for diabetes in adults [32]. The primary endpoint is HbA1c, measured 12 months (300 to 420 days) after enrolment. Secondary and exploratory endpoints are provided in Table 1 below. For all endpoints measured after 6 months, a window of 150 to 240 days and for 12 months’ endpoints, a window of 300 to 420 days after enrolment applies.

**Table 1:** Primary, secondary, and exploratory endpoints

**Primary endpoint**
• Mean HbA1c 12 months after enrolment
**Secondary endpoints**
• 10-year risk for a fatal or non-fatal cardiovascular event estimated using the WHO cardiovascular disease risk prediction tool [33] 6 and 12 months after enrolment • Mean HbA1c 6 months after enrolment • Mean fasting blood glucose (FBG) 6 and 12 months after enrolment • Proportion of participants with an HbA1c < 8% 6 and 12 months after enrolment • Proportion of participants with an FBG < 7 mmol/l 6 and 12 months after enrolment • CVD risk factors, such as BMI, abdominal circumference, blood lipid status, physical activity using the validated International Physical Activity Questionnaire Short Form (IPAQ-SF) [34], dietary habits using a shortened unquantified food frequency questionnaire adapted from an assessment tool for obesity used in South Africa [35], and alcohol and tobacco use 6 and 12 months after enrolment • Linkage to care: proportion of participants not taking treatment at enrolment who have initiated pharmacological antidiabetic treatment 6 and 12 months after enrolment • Engagement in care: proportion of participants who are engaged in care, defined as reporting intake of antidiabetic medication as per prescription of a healthcare provider (VHW or healthcare professional) 6 and 12 months after enrolment or reaching treatment targets without intake of medication • Occurrence of serious adverse events (SAEs) and adverse events of special interest (AESIs) within 6 and 12 months after enrolment • Self-reported adherence to antidiabetic treatment 6 and 12 months after enrolment
**Exploratory endpoints**
• Quality of life using the EQ-5D-5L instrument [36] and diabetes distress using the five item version of the "Problem Area in Diabetes" (PAID-5) Scale [37, 38] after 6 and 12 months and health beliefs using the Beliefs about Medicines Questionnaire adapted for people living with T2D [39, 40] after 12 months • Self-reported access to care and access to medication • Number of consultations at a health facility and with the VHW within 6 and 12 months after diagnosis • Trajectory of participants between facility-based and community-based care in the intervention villages (i.e. number of participants accepting community-based care at baseline, number of people switching to facility-based care and back to community-based care during the study period) • Proportion of participants with T2D who stop drug treatment or interrupt drug treatment for more than 3 weeks or require a switch of drug treatment due to (perceived) adverse events (AEs) within 6 and 12 months after enrolment • Proportion of participants who are reaching treatment targets (FBG < 7 mmol/l) and are reporting no intake of antidiabetic medication in the 2 weeks prior to assessment after 6 and 12 months • Proportion of participants accessing lipid-lowering medication 6 and 12 months after enrolment • Participants’, VHWs’, and involved healthcare professionals’ perception of the risks, benefits, and problems of community-based management of uncomplicated T2D by VHWs • Causes for the stop or interruption of treatment or switch to health facility-based treatment after initiation by VHWs in the community • Health system costs and individual costs for participants for the management of their condition within the first 6 and 12 months after diagnosis • 10-year CVD risk estimated using the Globorisk score [41] and Framingham Risk Score [42] 6 and 12 months after enrolment • Type and dosage of antidiabetic and lipid-lowering medications prescribed by VHWs or healthcare professionals 6 and 12 months after enrolment

*CVD* cardiovascular disease, *WHO* World Health Organization, *FBG* fasting blood glucose, *BMI* body mass index, *VHW* village health worker, *SAE* serious adverse event, *AESI* adverse event of special interest

Adverse events of special interest (AESIs) are defined as adverse events (AEs) consistent with T2D complications, such as stroke, myocardial infarction, hyperglycemic emergency, new diagnosis of heart failure, chronic kidney disease, blindness, diabetic foot syndrome, and AEs probably related to intake of antidiabetic medication, such as significant hypoglycemia (< 3 mmol/l and symptoms of hypoglycemia) and intolerance reactions against antidiabetic medication leading to discontinuation of the medication concerned (including allergic reactions, drug interactions, or other side effects).

### Measurements

Baseline and endpoint assessments except HbA1c measurements are conducted by VHWs guided by the ComBaCaL app through instructions for correct sample collection and structured questionnaires for the assessment of lifestyle risk factors, AESIs, SAEs, health beliefs, diabetes distress, and quality of life. HbA1c is collected by the study staff not directly involved in the intervention.

**Fig. 1:**
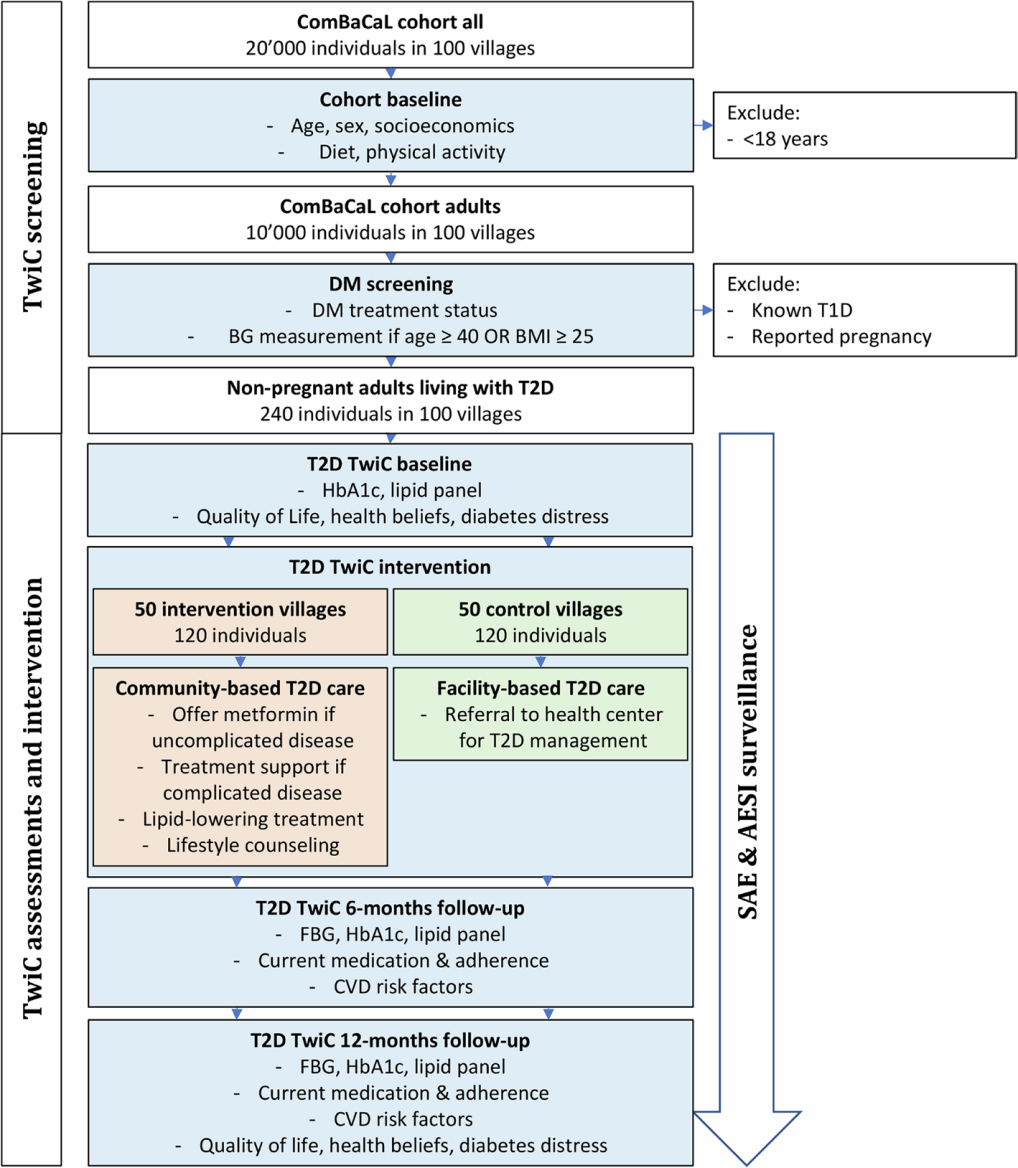
Flow of events. FBG, fasting blood glucose; CVD, cardiovascular disease; SAE, serious adverse event; AESI, adverse event of special interest; T2D, type 2 diabetes; T1D, type 1 diabetes

Most baseline data are extracted from the ComBaCaL cohort database, including anthropometrics, sociodemographic characteristics, targeted medical history, HIV status, cardiovascular complications, physical activity using the validated International Physical Activity Questionnaire Short Form (IPAQ-SF) [34], dietary habits using a shortened unquantified food frequency questionnaire adapted from an assessment tool for obesity used in South Africa [35], and self-reported alcohol and tobacco use (see Fig. 1 and Table 2). In addition to the cohort data, further baseline information, including HbA1c, blood lipids, quality of life using the EQ-5D-5L instrument [36], health beliefs using the Beliefs about Medicines Questionnaire (BMQ) adapted for people living with T2D [39, 40], and self-reported access to care are collected at TwiC enrolment.

**Table 2:** SPIRIT figure

**Time point**	** − 100–0** Cohort baseline	**0** TwiC baseline	**150–240** 6-month follow-up	**300–420** 12-month follow-up
**ComBaCaL cohort activities**				
ComBaCaL cohort informed consent^a^	X			
Date of birth	X			
Height, weight, abdominal circumference	X		X	X
Short medical history^b^	X			
CVDRFs^c^	X		X	X
T2D Screening^d^	X			
**TwiC assessments**				
FBG		X	X	X
HbA1c		X	X	X
Diabetes distress^e^			X	X
Health beliefs^f^		X		X
Quality of life^g^		X	X	X
Self-reported access to care and to medication		X	X	X
Blood lipid status^h^		X	X	X
Current antidiabetic and lipid-lowering medication	X	X	X	X
Adherence to antidiabetic medication		X	X	X
Screening for relevant clinical events		X	X	X
Screening for clinical alarm signs/symptoms		X	X	X
**TwiC control**				
Referral to health facility if required		X	X	X
**TwiC intervention**				
Offer metformin^i^		X	X	X
Offer lipid-lowering treatment^i^		X	X	X
Provide lifestyle counselling		X	X	X
Provide treatment support^j^		X	X	X
Referral to health facility^k^		X	X	X

*CVDRF* cardiovascular disease risk factor, *T2D* type 2 diabetes, *FBG* fasting blood glucose, *TwiC* trial within cohort, *LDL* low-density lipoprotein, *HDL* high-density lipoprotein

^a^Including consent to participation in TwiCs

^b^Including personal and family history for T2D

^c^Physical activity using IPAQ-SF [34], dietary habits [35], and tobacco and alcohol use

^d^According to Lesotho Standard Treatment Guidelines [31]

^e^Using the 5-item Problem Areas in Diabetes (PAID-5) scale [37, 38]

^f^Using the Beliefs about Medicines Questionnaire [39, 40]

^g^Using the EQ-5D-5L instrument [36]

^h^Total cholesterol, LDL, HDL, and triglycerides

^i^To participants eligible according to Lesotho Standard Treatment Guidelines [31]

^j^To participants receiving treatment from health facility (i.e. participants using insulin or more than one oral antidiabetic drug)

^k^In case of insufficient blood sugar control or clinical alarm symptoms

Endpoint assessments through home visits by VHWs for secondary endpoints and by study staff for HbA1c measurements are scheduled 6 months (range 150 to 240 days) and 12 months (300 to 420 days) after TwiC enrolment. During follow-up visits, VHWs in both groups inquire about the occurrence of possible SAEs or AESIs and document them in the ComBaCaL app. In addition, VHWs may solicit AESIs and SAEs through reporting by participants, friends, or relatives; screening of participants’ “bukanas” (personal health booklet); and reporting by routine health facility staff any time during the follow-up period.

Possible AESIs and SAEs flagged by the VHWs will be followed up by the supervising study staff to collect further clinical information (see Fig. 1 and Table 2). The pseudonymized reports will be submitted to the study physician who will remain blinded to the allocation. The study physician will classify the reports as SAEs, AESIs, or neither of the two and conduct a causality assessment for events classified as SAEs or AESIs. In addition, specific questionnaires about participants’ satisfaction with and acceptability of the TwiC intervention will be administered and semi-structured interviews conducted with a selection of participants, VHWs, and involved healthcare professionals to qualitatively explore perceived risks, benefits, problems, and acceptability of community-based therapeutic management of uncomplicated T2D.

### Statistical analysis and sample size

We will use different analysis sets as defined in Table 3. The primary analysis set will include all study participants with uncomplicated, uncontrolled T2D not requiring direct referral to facility-based care following the primary hypothesis and trial estimand for which we powered our sample size calculation. Uncontrolled uncomplicated T2D is defined as having a baseline fasting blood glucose (FBG) ≥ 7 mmol/l and taking no antidiabetic treatment or no more than one oral antidiabetic.

**Table 3:** Overview of analysis sets

	***Description***	***FBG***	***Treatment***	***Criteria for direct referral***	***HbA1c***	***Pregnancy***
*Primary analysis set*	Non-pregnant, uncomplicated, uncontrolled T2D, no direct referral required	≥ 7 mmol/l	No treatment or no more than one oral drug	Not met	Any	Non-pregnant
*Secondary analysis set a*	Non-pregnant, uncomplicated, uncontrolled T2D, no direct referral required, HbA1c ≥ 6.5%	≥ 7 mmol/l	No treatment or no more than one oral drug	Not met	≥ 6.5%	Non-pregnant
*Secondary analysis set b*	Non-pregnant T2D, HbA1c ≥ 6.5%	Any	Any	Any	≥ 6.5%	Non-pregnant
*Secondary analysis set c*	Non-pregnant T2D	Any	Any	Any	Any	Non-pregnant

Criteria for direct referral: FBG > 14 mmol/l or RBG > 16.7 mmol/l or having polyuria, polydipsia, and weight loss independent of blood glucose values

*T2D* type 2 diabetes, *FBG* fasting blood glucose, *RBG* random blood glucose

The criteria for direct referral to a health facility are FBG > 14 mmol/l or random blood glucose (RBG) > 16.7 mmol/l or having polyuria, polydipsia, and weight loss independent of blood glucose values. As sensitivity analyses, we will assess the primary and secondary endpoints in several secondary analysis sets as outlined in Table 3.

The sample size for this TwiC was calculated assuming an individual randomization inflated by a design effect that accounts for variation at cluster level, according to the code developed by Rotondi and Donner [43]. Based on preliminary results from an NCD prevalence survey in Lesotho [20], we expected the prevalence of T2D in the adult population in the rural setting in Lesotho to be approximately 4%, with about 60% of people living with T2D fulfilling the criteria for the primary analysis set (non-pregnant, uncomplicated and uncontrolled T2D, no direct referral required). Considering an average cluster size of 100 adult inhabitants, the mean number of inhabitants eligible for the TwiC is 2.4 per village. We estimated a clinically significant effect size of 0.6% HbA1c mean difference between the two groups after 12 months. Assuming an intra-cluster correlation of 0.015 and an attrition rate of 20%, we calculated that a sample size of 240 individuals or 100 villages (120 per arm, 50 villages per arm) is required to detect superiority with a type I error of 0.05 and a statistical power of 80%. We will not be able to include more villages if the sample size is not reached. To ensure optimal recruitment in the ComBaCaL villages, all potential participants will be regularly visited at home for screening and offer of the intervention. Analyses will be performed following the principles for analysis of cluster randomized trials in health research as outlined by Donner and Klar [44]. We will consider blinding the statistician for the primary endpoint analysis. All analysis sets will be analysed according to the intention-to-treat principle, i.e. all participants will be analysed in the groups to which they were randomized. We will use a linear mixed-effect regression model with a random intercept for clusters and adjust for the prespecified stratification factors and potentially unbalanced confounders between the groups. Statistical significance will be based on 2-sided tests at the alpha level of 0.05. No traditional per-protocol or as-treated analyses are planned since they assume completely random compliance patterns. Instead, we plan appropriate complier average causal effect analyses to account for non-compliance with the intervention [45]. Secondary endpoints will be reported using descriptive statistics such as the mean and 95% Wald confidence intervals, frequency, and percentages. Participants with missing covariates will be imputed using multiple imputation-chained equation techniques. Further details will be outlined in a statistical analysis plan.

### Data management and monitoring

Each VHW in the ComBaCaL cohort study received a password-protected tablet with the ComBaCaL app installed. The ComBaCaL app is based on the open-source Community Health Toolkit Core Framework, a widely used, offline-first, open-source software toolkit designed for community health systems [46]. Data will be synchronized regularly to a secure server hosted at the University Hospital Basel. Data are monitored locally by the VHW supervisors and centrally by the data management team of the University Hospital Basel. All data exports will be pseudonymized. The intervention assessed in this TwiC entails the task-shifting of basic T2D services according to local and international guidelines. It has a low risk profile and therefore neither the establishment of a data monitoring committee nor a formal interim analysis nor external auditing is planned.

## Discussion

Many countries in sub-Saharan Africa and other LMICs have established VHW systems that are traditionally focusing on maternal and neonatal health and on communicable diseases, especially HIV/AIDS [47]. In recent years, increasing evidence has emerged showing a beneficial effect and high cost-effectiveness of VHW-based models for diseases outside the traditional scope, especially for NCDs [11, 12, 15, 48–50]. However, for VHW-led T2D care models in sub-Saharan Africa, the evidence remains limited [18]. Most research studies assessing VHW-based T2D care models focused on educational [51, 52], screening and referral services [53], or self-management support [14] while it remains unexplored whether VHWs may safely and effectively deliver active treatment initiation and monitoring [54]. In South Africa, NCD screening by VHWs at the community level has proven effective for the detection of new T2D cases [53]. However, only 29% of participants with elevated blood glucose identified during screening linked to facility-based care after referral by VHWs, indicating limited effectiveness of community-based screening and referral services alone [53]. Considering these results, the remoteness of many Lesotho villages with difficult access to regular facility-based care and the successful experiences of VHWs providing HIV testing services at the community level in the same setting [25], we are proposing a model of care which capacitates VHWs to provide comprehensive community-based T2D services including first-line drug prescription, in addition to the screening, diagnostic, and counselling services that have been tested previously.

Using mobile health applications to improve T2D care outcomes has been explored extensively with promising results mainly in high-income settings [13, 55–57]. The large majority of digitally supported T2D interventions have used tools directly addressed to patients providing educational content, behavioural interventions, or remote consultations by healthcare professionals [56–58]. However, such approaches are difficult to implement in settings where access to mobile devices as well as digital and health literacy are limited. In such settings, digital tools with a clinical decision support component to guide VHWs providing services to patients seem more promising, especially if functional VHW systems are already in place. The use of a digital CDSS may enable more complex services by VHWs through algorithmic guidance and efficient real-time remote supervision. In their guidelines on digital interventions for health system strengthening, the World Health Organization is thus recommending the use of mobile CDSS for health workers at the community level [59].

Cardiovascular disease is the main cause of death among people living with T2D, and all international treatment guidelines recommend a comprehensive approach to cardiovascular risk factor control for T2D patients [60]. Multifactorial interventions tackling relevant risk factors have proven highly effective [61], and the feasibility of providing such interventions at the community level has been demonstrated in other settings [62] while such evidence is lacking for sub-Saharan Africa. Hence, our intervention not only includes glycaemic control measures alone but consists of a comprehensive package including lifestyle counselling and lipid-lowering and antiplatelet treatment for those eligible, and the estimated 10-year risk for a cardiovascular event is a key secondary outcome. Furthermore, we aim to explore integration with services for other chronic diseases such as arterial hypertension in similar TwiCs (NCT05684055) within the ComBaCaL cohort study.

In summary, this trial is assessing the feasibility and effectiveness of a comprehensive, CDSS-supported, setting-adapted, community-based T2D intervention within the existing Lesotho Village Health Program. It will generate the evidence required for the future development of community-based chronic disease care models in Lesotho and other countries with a similar VHW programme.

## Trial status

Recruitment for the TwiC started on May 13, 2023. A total of 226 participants were enrolled at the date of the revised submission of this manuscript on October 06, 2023. We expect recruitment to be completed by December 2023.

### Supplementary Information

**Additional file 1.**

## Data Availability

We will make pseudo-anonymized individual data freely available on a suitable repository, such as zenodo.org, along with the publication of the study results. The full protocol as submitted to the ethics committees is available on ClinicalTrials.gov. The study results will be published in a peer-reviewed journal without the use of professional writers and communicated to local health authorities and community stakeholders. Access to the test environment of the ComBaCaL app will be available from the corresponding author upon reasonable request.

## Abbreviations

AESI: Adverse event of special interest
BMI: Body mass index
CDSS: Clinical decision support system
ComBaCaL: Community-Based chronic disease Care Lesotho
CVDRF: Cardiovascular disease risk factor
FBG: Fasting blood glucose
HbA1c: Glycated haemoglobin
HDL: High-density lipoprotein
LDL: Low-density lipoprotein
LMICs: Low- and middle-income countries
NCD: Non-communicable disease
PAID-5: Problem Areas in Diabetes 5-Item Questionnaire
RBG: Random blood glucose
SAE: Serious adverse event
TwiC: Trial within cohort
T1D: Type 1 diabetes
T2D: Type 2 diabetes
VHW: Village health worker
WHO: World Health Organization
